# Alignment System and Application for a Micro/Nanofluidic Chip

**DOI:** 10.3390/mi9120621

**Published:** 2018-11-24

**Authors:** Junyao Wang, Lu-lu Han, Ye-ming Sun, Tian-yi Su

**Affiliations:** 1 School of Mechanical Engineering, Northeast Electric Power University, Jilin 132012, China; 2201600344@neepu.edu.cn (L.-l.H.); 20142557@neepu.edu.cn (T.-y.S.); 2 School of Mechanic Engineering, Jilin University, Changchun 130000, China

**Keywords:** alignment system, micro/nanochannel, micro/nanofluidic, chip fabricating, ion enrichment

## Abstract

In this paper, a direct pre-bonding technology after alignment of the chip is presented to avoid the post-misalignment problem caused by the transferring process from an alignment platform to a heating oven. An alignment system with a high integration level including a microscope device, a vacuum device, and an alignment device is investigated. To align the chip, a method of ‘fixing a chip with microchannels and moving a chip with nanochannels’ is adopted based on the alignment system. With the alignment system and the assembly method, the micro/nanofluidic chip was manufactured with little time and low cost. Furthermore, to verify the performance of the chip and then confirm the practicability of the device, an ion enrichment experiment is carried out. The results demonstrate that the concentration of fluorescein isothiocyanate (FITC) reaches an enrichment value of around 5 μM and the highest enrichment factor is about 500-fold. Compared with other devices, an alignment system presented in this paper has the advantages of direct pre-bonding and high integration level.

## 1. Introduction

A microfluidic technology [1] called lab-on-a-chip with the main feature of operating fluid on a micron scale can achieve the function of a chemistry and biology lab in several square centimeters [2,3,4,5]. However, the efficiency of biochemical reactions as a result of employing microfluidic technology is still not ideal. To improve the property of the microfluidic technology, the nanostructure is embedded into the chip with microchannels forming a micro/nanofluidic chip suitable for single molecule detection, separation analysis, and protein enrichment due to the size of the nanochannels, which can accommodate biological macromolecules such as DNA and proteins. Nevertheless, the chip’s small size has many advantages such as a rapid reaction rate [6,7], low cost, and high efficiency. It has not been promoted widely owing to the associated complicated processes of photolithography, development, micro-manufacturing, nano-manufacturing, and micro/nano integration [8,9,10]. Notably, micro/nano integration manufacturing is the bottleneck of the micro/nanofluidic chip’s development because of the high-accuracy requirement and the time needed to process the chips.

Traditionally, two methods for manufacturing the microfluidic chip are utilized. One method integrates micro/nanochannels in a chip, which requires highly accurate photolithography and overlay [11]. Fortunately, the other method fabricates microchannels and nanochannels in two chips and compensates for the former method’s insufficiency [12]. Few devices have met the needs of the market identified herein. For instance, an alignment device was established by Fuentes et al. [13] for a microfluidic chip composed of multi-layer polymethyl methacrylate (PMMA). Microchannels were aligned from top to bottom with holes in the middle layer. Their multilayered microfluidic chips in thermoplastic polymers made fabrication easier and faster. Nevertheless, the device can not accomplish the operation process of the micro/nano dimension. Fortunately, a micro-assembly work cell developed by Zong et al. [14] achieved the micro/nano dimension operation based on the critical technologies including precise positioning, state maintenance, and system integration. However, in order to realize the alignment process, a method with the auxiliary markers must be adopted. A robotic micro-assembly device proposed by David et al. [15] developed the assembly of micro-objects on a large substrate without the markers. Nonetheless, the horizontal rotation movement was not integrated into that device. A multichip alignment device presented by Chen et al. [16] implemented multiple alignment bonding at the same time on the basis of a centrifugalization alignment technology, whereas the chips must be fixed stack and transferred to the bonding chamber for the bonding. Up to this point, no literature about an alignment system with the advantages of direct pre-bonding and high integration level has been reported.

In this paper, an alignment system with a pre-bonding device for fabricating a micro/nanofluidic chip is demonstrated and an assembly method referred to ‘fixing a chip with microchannels and moving a chip with nanochannels’ is adopted. Based on this system, a micro/nanofluidic chip is manufactured and an ion enrichment experiment is developed and the experiment results are analyzed. In the end, the performance of the alignment system compared with other devices is discussed.

## 2. Alignment System

An alignment system for a micro/nanofluidic chip is composed of a microscope device, a vacuum device, and an alignment device. A position relationship between a chip with nanochannels and a chip with microchannels is observed constantly through utilizing a microscope unit and adjusting with a vacuum unit, adsorbing a chip with nanochannels in the process of alignment. Ultimately, an alignment operation for a chip with nanochannels and a chip with microchannels is achieved with an alignment unit adjusting a three-coordinate position and an angle between a microstructure and a nanostructure.

### 2.1. Microscope Device

An alignment process for micro/nanochannels is observed and then a real-time image is obtained through adopting the microscope unit constituting mainly of an inverted fluorescence microscope (OLYMPUS IX73, Olympus Corporation, Tokyo, Japan), a CCD (charge coupled device) camera, and a real-time imaging software. There are two advantages for the system. Firstly, the micro/nanostructure can be viewed clearly in real time for an equipment’s efficiency pixel of 2 million. Secondly, an external device can be integrated due to a sufficient operating space provided by an inverted fluorescence microscope.

### 2.2. Vacuum Device

**Figure 1:**
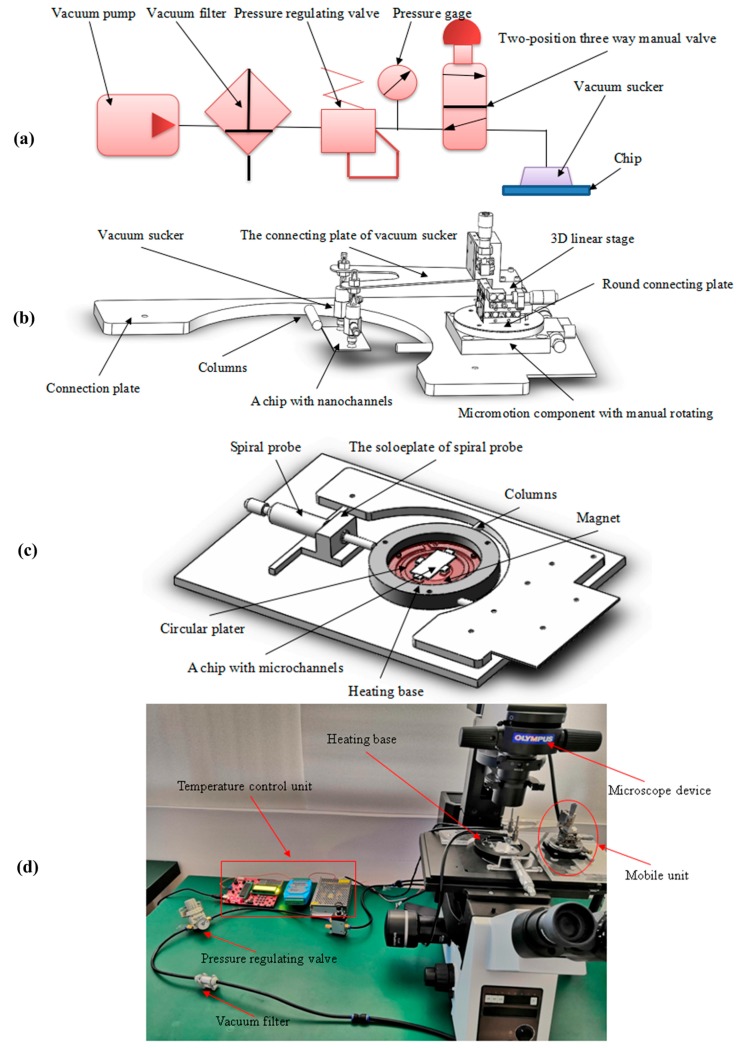
The alignment system for a micro/nanofluidic chip. (**a**) The vacuum device including a vacuum pump, a pressure regulating valve, and other components is adopted to realize the function of air path control and adsorb the chip with nanochannels through the vacuum sucker. (**b**) The mobile unit integrated with a 3D linear stage, a micromotion component with manual rotating, and other parts are employed to develop the movement of the chip with the nanochannels. The position adjustment of the chip with the nanochannels in the three-dimensional direction and horizontal direction is completed by adjusting the screw micrometer head. (**c**) The fixed unit composed of a screw micrometer, a spiral probe, and other parts is utilized to achieve the fixation of the chip with the microchannels. The heating base and the circular plater are introduced to implement the pre-bonding of the chip based on an ohmic heating principle. (**d**) The real images of the set up. The pre-bonding temperature is 60 °C for 2 h through regulating temperature control unit.

A chip with nanochannels is adsorbed via employing a vacuum device primarily made up of a vacuum pump, a vacuum filter, a pressure regulating valve, and a two-position three-way manual valve, as shown in Figure 1a. A working principle for the vacuum system is as follows: a chip with nanochannels is adsorbed due to producing a vacuum in the vacuum sucker by means of switching on a vacuum pump, adjusting a pressure regulating valve, and then unfolding a two-position three-way manual valve. When completing an alignment operation, a chip with nanochannels is ultimately separated from the vacuum sucker through shutting down a two-position three-way manual valve to remove the vacuum from the vacuum sucker.

### 2.3. Alignment Device

The alignment regulatory device is divided into two parts including a mobile unit for adjusting the position of a chip with nanochannels and a fixed unit for fixing a chip with microchannels. The mobile unit mainly consists of a micromotion component with manual rotation, a 3D linear stage, and the vacuum suckers in Figure 1b. The range and accuracy of the micromotion component with manual rotation are 0–6.5 mm and 0.01 mm, respectively, in directions of *x*, *y,* and *z* axis. The coarse, fine, and precision tuning of a micromotion component are 0–360°, 0–6.5°, and 0.0167°, respectively.

A fixed unit is mainly made up of a chip container, four pairs of magnets sized 10 × 5 × 2 mm, and a spiral probe with the measuring range of 0–25 mm in Figure 1c. A modulating spiral probe enables close contact between chip container and supporting columns, which immobilizes the chip container. Through arranging the position of the four pairs of magnets distributed on the upper and lower surfaces of the chip container, close contact between the magnets and the chip is developed to fix a chip with microchannels in the chip container. Figure 1d demonstrates that the heating base and the circular plater is introduced to implement the pre-bonding of the chip based on an ohmic heating principle. After which, the pre-bonding temperature is 60 °C for 2 h through regulating temperature control unit.

## 3. Assembly Method

The method of ‘fixing a chip with microchannels and moving a chip with nanochannels’ was adopted for the assembly of a micro/nanofluidic chip based on a micro-image technique. The specific steps are as follows.

The first step—fixing a chip with microchannels. Firstly, a chip container is in a stage of the inverted fluorescence microscope and connects with two supporting columns. Then, via close contact between the chip container and supporting columns, modulating a spiral probe is carried out to immobilize the chip container. Secondly, a chip with microchannels is situated in the chip container, and four pairs of magnets are placed at the end face of the chip with microchannels. Lastly, the chip with microchannels is steadied by means of modulating the position of magnets. The advantages of this method are simple, low cost, and free from size and shape.

**Figure 2:**
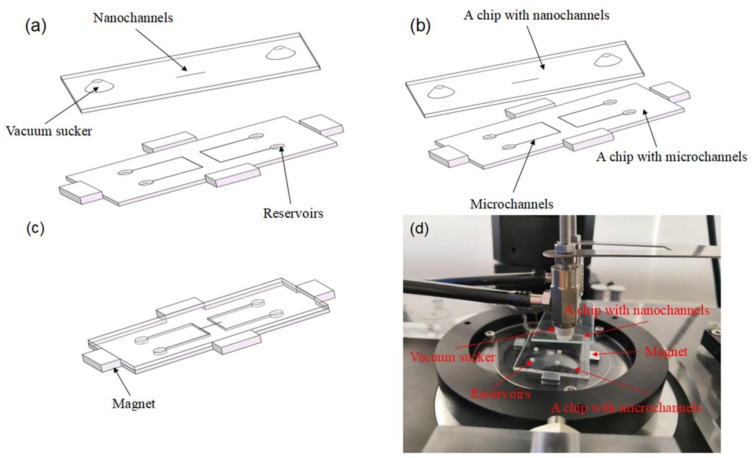
The assembly process for the micro/nanochannels. (**a**) The chip with the microchannels is fixed through utilizing four pairs of magnets, and the chip with the nanochannels is adsorbed through the vacuum sucker. Four reservoirs are employed to fill the solution into the channel. (**b**) An angle position of the micro/nanochannels is insured by means of regulating a screw’s micrometer connecting to micromotion component with manual rotation. (**c**) The position for *x*, *y*, and *z* axes is viewed based on the imaging system of the inverted fluorescence microscope via regulating a screw’s micrometer of a 3D linear stage. (**d**) The real images of the set up.

The second step—moving a chip with nanochannels. Firstly, the chip with nanochannels is adsorbed due to producing vacuum in the vacuum sucker through successively opening a vacuum pump, a vacuum filter, and a two-position three-way manual valve, as shown in Figure 2a. Secondly, an angle position of micro/nanochannels is insured by means of regulating a screw’s micrometer connecting to micromotion component with manual rotation, as shown in Figure 2b. Thirdly, the position for *x*, *y*, and *z* axes is viewed based on the imaging system of the inverted fluorescence microscope via regulating a screws micrometer of a 3D linear stage as shown in Figure 2c,d, which demonstrates the real images of the set up.

A new design concept is provided from existing business methods. A flip-chip bonding technique is employed to link metal bump directly to wire substrate [17]. However, due to channel blocking resulted from metal bump meltdown, micro/nanofluidic chip’s dimensional inconsistency, and incomplete connection or sealing, this method does not apply to the bonding of a plastic chip integrated with micro/nanochannels. In addition, a pick and place technique is widely employed in a porter robot field [18]. This method is similar to the method in this paper; a chip is picked up and then another chip is placed. Nevertheless, higher accuracy, better judgment, and finer operation are required for the micro/nanochannel’s movement and alignment in the micro/nano scale.

The method utilized in this paper is distinguished from others in the following two aspects. On the one hand, due to micro/nanofluidic chip’s dimensional inconsistency and micro/nanochannel’s position uncertainty, to recognize the micro/nanochannel’s relative position raises new requirements for an alignment method. Therefore, a method of ‘fixing a chip with microchannels and moving a chip with nanochannels’ assisted with an inverted fluorescence microscopy is employed for real-time observation and treatment. To save more space, operate more easily, and promote higher integration level, on the other hand, an alignment system integrated with the functions including three-dimensional moving and angle rotating is adopted.

## 4. Results and Discussion

### 4.1. Application Experiment of Alignment System

**Figure 3:**
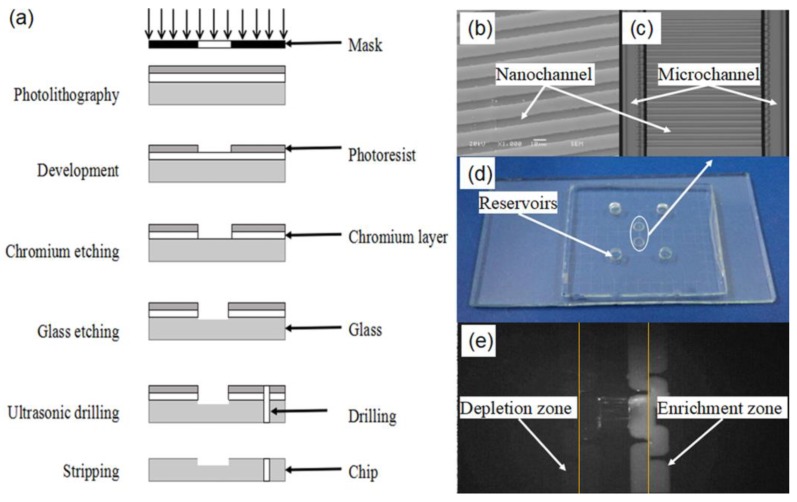
Application experiment of alignment system. (**a**) The manufacturing process of a glass micro/nanofluidic chip. (**b**) The scanning electron micrograph (SEM) photograph of the nanochannels before assembly. (**c**) The local enlarged drawing of micro/nanochannels. (**d**) The micro/nanofluidic chip. (**e**) Working sketches of ion enrichment experiments.

The micro/nano alignment and the pre-bonding are two significance steps of fabricating a glass chip, rather than overall process. To confirm the practicability of the device, detailed fabrication steps are introduced. The manufacturing process of a glass micro/nanofluidic chip is shown in Figure 3a, as follows. (1) A photolithography is employed to expose for about 60 s on a lithography machine. Next, a photoresist pattern and a chromium pattern are obtained through the 0.6~1% NaOH developing solution and the dechromization liquid, respectively. (2) The microchannels and the nanochannels are fabricated by means of adopting a mixed solution including hydrofluoric acid, nitric acid, and deionized water (at a ratio of 5:10:85). (3) The end of the microchannels is fabricated using an ultrasonic drilling machine, then the chromium layer and the photoresist layer on the glass surface are removed. (4) The microchannels and the nanochannels exhibited in Figure 3b on the glass are aligned through the device presented in this paper. (5) A hot plate and a muffle furnace are employed to pre-connect at 60 °C for 2 h and bonded under the highest bonding temperature of 580 °C for 12 h. The micro/nanofluidic chip consists of a chip with nanochannels and a chip with microchannels developed by adopting the device. In addition, the microchannels are configured in an array in Figure 3c. These have a width of 200 μm and a depth of 30 μm, have a double U-shape, and 20 nanochannels with a width of 5 μm and a depth of 200 nm. Ultimately, the U-shaped microchannels are connected through the nanochannels, as shown in Figure 3d.

Ion enrichment experiments are carried out based on the micro/nanofluidic chip. Firstly, the reservoirs, the microchannels, and the nanochannels on the chip are filled with a solution of 10 nmol/L FITC (fluorescein isothiocyanate). Afterwards, four platinum electrodes are inserted one at a time into the reservoir, and a voltage of 300 V is applied. In the next place, based on the inverted fluorescence microscope, the enrichment occurs at the interface of the microchannels and the nanochannels, as shown in Figure 3e. At the interface of anodes, a significant depletion of ions occurs. Conversely, an obviously enrichment of ions appears around the interface of the microchannels and the nanochannels where enrichment increases with increased time.

**Figure 4:**
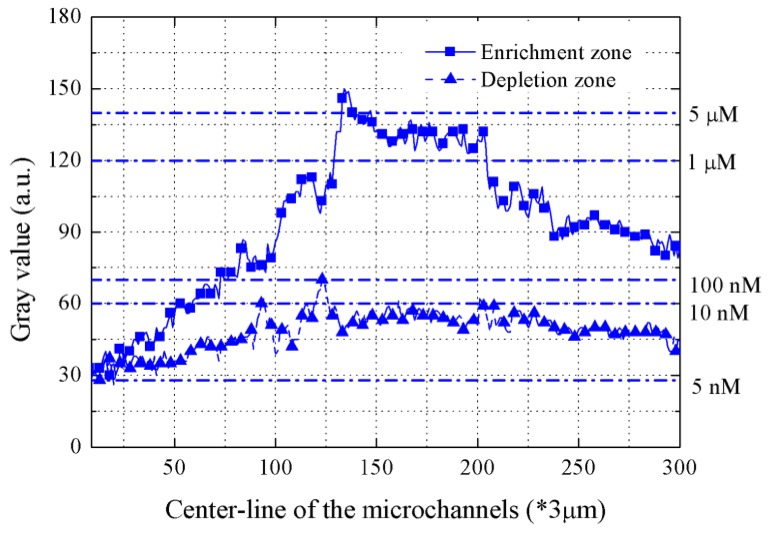
Plot of FITC fluorescence intensity with initial concentration of 10 nM in the center-line of the microchannels.

Figure 4 demonstrates the plot of FITC strength with initial concentration of 10 nM with the applied voltage of 300 V. It is seen that the enrichment strength of FITC sample in the enrichment zone is higher than that in the depletion zone. Under an applied electric field, cations transport from the anode to the cathode and are able to pass through the nanochannels, while anions are difficult to across that for exclusion-enrichment effect of the nanochannel’s double electrode layer (EDL). In conclusion, Anions are enriched at micro/nano zone and the FITC enrichment occurs. Simultaneously, it can be known that the enrichment strength of FITC near the nanochannels is higher than that far from the nanochannels from the plot of the enrichment zone. Specifically, the concentration of FITC reaches an enrichment value of around 5 μM. The highest factors for the enrichment and the depletion are about 500-fold and 2-fold, respectively.

### 4.2. Comparing Microassembly Devices for Chip

From the Table 1, the accuracy of all devices reaches to micrometers. Among these, the accuracy of the micro-assembly work cell is the highest, whereas the operating dimension is not at the micro/nano scale [14]. Consequently, the micro-assembly work cell is not applied to the alignment system in this paper. On the one hand, the range of the alignment system is just 6.5 mm relative to the other devices with no constraint from the range. On the other hand, all kinds of transparent material can be aligned by the alignment system. Nonetheless, the glass chips are not suitable for the microfluidic device which is mainly aimed at the alignment of multi-layer polymer material chips [13]. Additionally, the operating space of the micro-assembly work cell is larger than the other cells. Nevertheless, only PMMA chips are appropriated for the micro-assembly work cell, and two markers on the diagonal line of the substrate and the cover must be employed simultaneously to achieve the alignment operation [14]. After the alignment, the bonding process is a critical step. A phase transition sacrificial layer assisted by solvent bonding is employed to achieve the chip bonding [13], meanwhile, a hot fusion method is utilized to bond the substrate and the cover through a set of heating components [14]. Fortunately, a direct pre-bonding technology after the alignment of the chip in this paper is presented to avoid the post-misalignment problem caused by a transferring process from an alignment platform to a heating oven. In summary, the alignment system meeting all the requirements in this paper has advantages of employing a direct pre-bonding technology, applied to a micro/nano field, and utilizing an unrestricted chip material.

## 5. Concluding Remarks

An alignment system with a pre-bonding device for fabricating a micro/nanofluidic chip is demonstrated and an assembly method referred to ‘fixing a chip with microchannels and moving a chip with nanochannels’ is adopted. With the alignment system and the assembly method, the micro/nanofluidic chip was manufactured. To verify the performance of the chip and then confirm the practicability of the device, a performance experiment of ion enrichment was realized successfully. The enrichment experiment results demonstrate that the concentration of FITC reaches an enrichment value of around 5 μM. Specifically, the highest factors for the enrichment and the depletion are about 500-fold and 2-fold respectively. Compared with other devices, an alignment system presented in this paper has the advantages of direct pre-bonding and high integration level. In summary, the alignment system meeting all the requirements in this paper has advantages of employing a direct pre-bonding technology, applied to a micro/nano field, and utilizing an unrestricted chip material.

## Figures and Tables

**Table 1:** Parameters comparison of different systems.

System	Number of Precision Regulators	Accuracy	Range	Chip Material	Operating Space	Marking Chip	Pre-Bonding Directly
[13]	One	0.026 mm	-	Polymer	Common	no	no
[14]	One	0.0029 mm	-	PMMA	Large (1750 × 300 × 150 mm)	yes	no
The Alignment System	One	0.02 mm	6.5 mm	Transparent Material	Common (170 × 80 × 105 mm)	no	yes
